# Mycobacterium *tuberculosis* thymidylate kinase antigen assays for designating incipient, high-risk latent M.*tb* infection

**DOI:** 10.1186/s12879-018-3007-y

**Published:** 2018-03-16

**Authors:** Misaki Wayengera, David P. Kateete, Benon Asiimwe, Moses L. Joloba

**Affiliations:** 1 Department of Pathology, Unit of Genetics & Genomics, School of Biomedical Sciences, Makerere University College of Health Sciences, P o Box 7072, Kampala, Uganda; 2 Department of Immunology &Molecular Biology, School of Biomedical Sciences, Makerere University College of Health Sciences, P o Box 7072, Kampala, Uganda; 3 Department of Medical Microbiology, School of Biomedical Sciences, Makerere University College of Health Sciences, P o Box 7072, Kampala, Uganda

**Keywords:** Tuberculosis, Mycobacterium *tuberculois*, Latent M*.tb* infections (LTBI);Thymidylate Kinase, Serodiagnosis

## Abstract

**Background:**

Precise designation of high risk forms of latent Mycobacterium *tuberculosis*-M*.tb* infections (LTBI) is impossible. Delineation of high-risk LTBI can, however, allow for chemoprophylaxis and curtail majority cases of active tuberculosis (ATB). There is epidemiological evidence to support the view that LTBI in context of HIV-1 co-infection is high-risk for progression to ATB relative to LTBI among HIV-ve persons. We recently showed that assays of M*.tb* thymidylate kinase (TMKmt) antigen and host specific IgG can differentiate ATB from LTBI and or no TB (NTB, or healthy controls). In this study, we aimed to expose the differential levels of TMKmt Ag among HIV+ve co-infected LTBI relative to HIV-ve LTBI as a strategy to advance these assays for designating incipient LTBI.

**Methods:**

TMKmt host specific IgM and IgG detection Enzyme Immuno-Assays (EIA) were conducted on 40 TB exposed house-hold contacts (22 LTBI vs. 18 no TB (NTB) by QunatiFERON-TB GOLD®); and TMKmt Ag detection EIA done on 82 LTBI (46 HIV+ve vs 36 HIV-ve) and 9 NTB (American donors). Purified recombinant TMKmt protein was used as positive control for the Ag assays.

**Results:**

IgM levels were found to be equally low across QuantiFERON-TB GOLD® prequalified NTB and TB exposed house-hold contacts. Higher TMKmt host specific IgG trends were found among TB house-hold contacts relative to NTB controls. TMKmt Ag levels among HIV+ve LTBI were 0.2676 ± 0.0197 (95% CI: 0.2279 to 0.3073) relative to 0.1069 ± 0.01628 (95% CI: 0.07385 to 0.14) for HIV-ve LTBI (supporting incipient nature of LTBI in context of HIV-1 co-infection). NTB had TMKmt Ag levels of 0.1013 ± 0.02505 (5% CI: 0.0421 to 0.1606) (intimating that some were indeed LTBI).

**Conclusions:**

TMKmt Ag levels represent a novel surrogate biomarker for high-risk LTBI, while host-specific IgG can be used to designate NTB from LTBI.

**Electronic supplementary material:**

The online version of this article (10.1186/s12879-018-3007-y) contains supplementary material, which is available to authorized users.

## Background

Tuberculosis (TB) is the leading infectious cause of death world-over. TB is caused by infection with the tubercle bacillus *Mycobacterium tuberculosis* (M. *tb*) [1, 2]. An estimated 1/3 of the global human population is thought to be infected with M.*tb.* Majority (90–95%) of these infections remain asymptomatic (a.k.a latent M.*tb* infection, LTBI) [3–5]. Only 10-5% progress to active TB (ATB) disease. In 2016, there were an estimated 1.3 million TB deaths among HIV-negative people (down from 1.7 million in 2000) and an additional 374 000 deaths among HIV-positive people. About 10.4 million people fell ill with TB in 2016: 90% were adults, 65% were male, 10% were people living with HIV (74% in Africa) and 56% were in five countries: India, Indonesia, China, the Philippines and Pakistan [3–5]. Hopes of totally controlling TB have been dampened because of the (i) difficulty of developing an effective vaccine, (ii) expensive and time-consuming diagnostic process, (iii) necessity of many months of treatment, (iv) increase in HIV-TB co-infections, and (iv) emergence of drug-resistant cases in the 1980s [3–5].

Because LTBI forms the vast reservoir from which ATB accrues, it has been proposed that identifying those incipient forms of LTBI that are at high-risk of progressing to ATB and treating or offering them chemo-prophylaxis, can drastically reduce global TB incidence [3, 5]. Accurate designation of high-risk or incipient LTBI is, *however,* currently impossible. Specifically, the two available methods (the tuberculin skin test-TST and interferon gamma release assays- IGRA) for testing for LTBI cannot designate high-risk LTBI. TST if positive provides evidence of M*.tb* infection. That said, many HIV infected patients will have a negative skin test despite M*.tb* infection or disease, due to anergy. “Two stage or booster test” is not a substitute to anergy testing. However, the same might have some utility in detecting M*.tb* infection in anergic HIV-TB co-infected patients [6]. TST underestimates the prevalence of LTBI in endemic countries, requires trained health care staff to correctly perform and accurately read the results, and also demands a second patient visit [7–13]. The test is neither useful to rule in disease nor in high TB prevalence settings to identify eligible individuals for prophylaxis. Interferon-γ release assay (IGRA) is used to diagnose LTBI and is particularly useful in profoundly ill patients and those with severe malnutrition. Two *in vitro* IGRA tests are available: QuantiFERON- TB Gold (Cellestis, USA) and the T SPOT-TB test (Oxford Immunotec, USA). Both use an enzyme- linked immunospot assay to quantify the number of peripheral blood mononuclear cells (PBMCs) producing IFN- γ in response to TB-specific antigen stimulation (ESAT-6 and CFP10). Both assays have sensitivity (as measured in patients with ATB) comparable to that of the TST, but are significantly more expensive [14]. IFN-γ assays do not differentiate between LTBI and ATB or between immune reconstitution inflammatory syndrome (IRIS) and failure. Studies suggest that IGRAs are ideal for serial testing because these can be repeated without boosting [15–17]. These are also unaffected by previous BCG vaccination and require fewer patient visits. However, WHO recommended against the use of IGRAs for diagnosis of active or latent TB, in resource-limited settings [18, 19].

There is epidemiologically evidenced high risk for acquiring ATB (over 20% per year) among HIV-1 co-infected persons [20]. This has been used to argue the incipient nature of LTBI among HIV+ve people and is the globally acceptable rationale underlying recommendations for isoniazid-INH chemo-prophylaxis. Overall, TB is the most common opportunistic infection (OI) among HIV-infected individuals, and co-infected individuals are at high risk of death [21–23]. TB may occur at any stage of HIV disease and is frequently the first recognized presentation of underlying HIV infection [24, 25]. As compared to people without HIV, people living with HIV (PLWH) have a 20-fold higher risk of developing TB [26, 27] and the risk continues to increase as CD4 cell counts progressively decline [24, 26, 27]. Although antiretroviral therapy (ART) can reduce the incidence of TB both at the individual and population level, PLWH on ART still have higher TB incidence rates and a higher risk of dying from TB [28, 29]. However, co-administration of ART along with anti-TB therapy presents several management challenges, including drug-drug interactions, overlapping drug toxicities and immune reconstitution syndrome. This emphasizes the importance of routine TB screening among PLWHA to not only identify those without TB, but if possible, prevent TB by chemoprophylaxis as well as to diagnose and promptly treat TB. In absence of accurate tests for high-risk LTBI, WHO recommends INH chemo-prophylaxis for all PLWHA [30–32].

Our group recently demonstrated the capability of TMKmt Ag and Ab assays to differentiate between ATBI and LTBI or NTB [33]. In the current study, we—riding on the evidence that LTBI among HIV-1 co-infected persons is high-risk, set out to examine LTBI diagnostic potential of TMKmt assays. Specifically, in light of the above described existing epidemiological data pointing to the high-risk of LTBI among HIV-1 co-infected persons to progress to ATB relative to their HIV-1 negative counterparts, it was argued that a point cross-sectional study of TMKmt Ag levels among HIV-ve LTBI relative to HIV-ve LTBI participants, can inform the diagnostic potential of TMKmt Ag assays for high risk LTBI. As a secondary outcome, we also examined the ability of TMKmt host specific IgM and IgG to differentiate between NTB and LTBI relative to the QuantiFERON-TB GOLD® assay.

## Methods

### Statement of ethics and consent to participate

This protocol was approved by the School of Biomedical Sciences Institutional Review & Ethics Committee (SBS-IREC) at the College of Health Sciences, Makerere University Kampala, Uganda as protocol # SBS 263 titled “Exploration of *Mycobacterium tuberculosis* thymidylate kinase based culture- and immunodiagnostic- assays towards rapid and easy detection of Tuberculosis”. Since the study used broadly consented serum of TB house-hold contacts and LTBI previously collected by the Makerere University-Case-Western Reserve University (MU-CWRU) TB Research Unit Project, the need for participant consent was waived by the SBS-IREC.

### TMKmt Peptide-epitopes and polyclonal antibodies (PAbs)

**Fig. 1:**
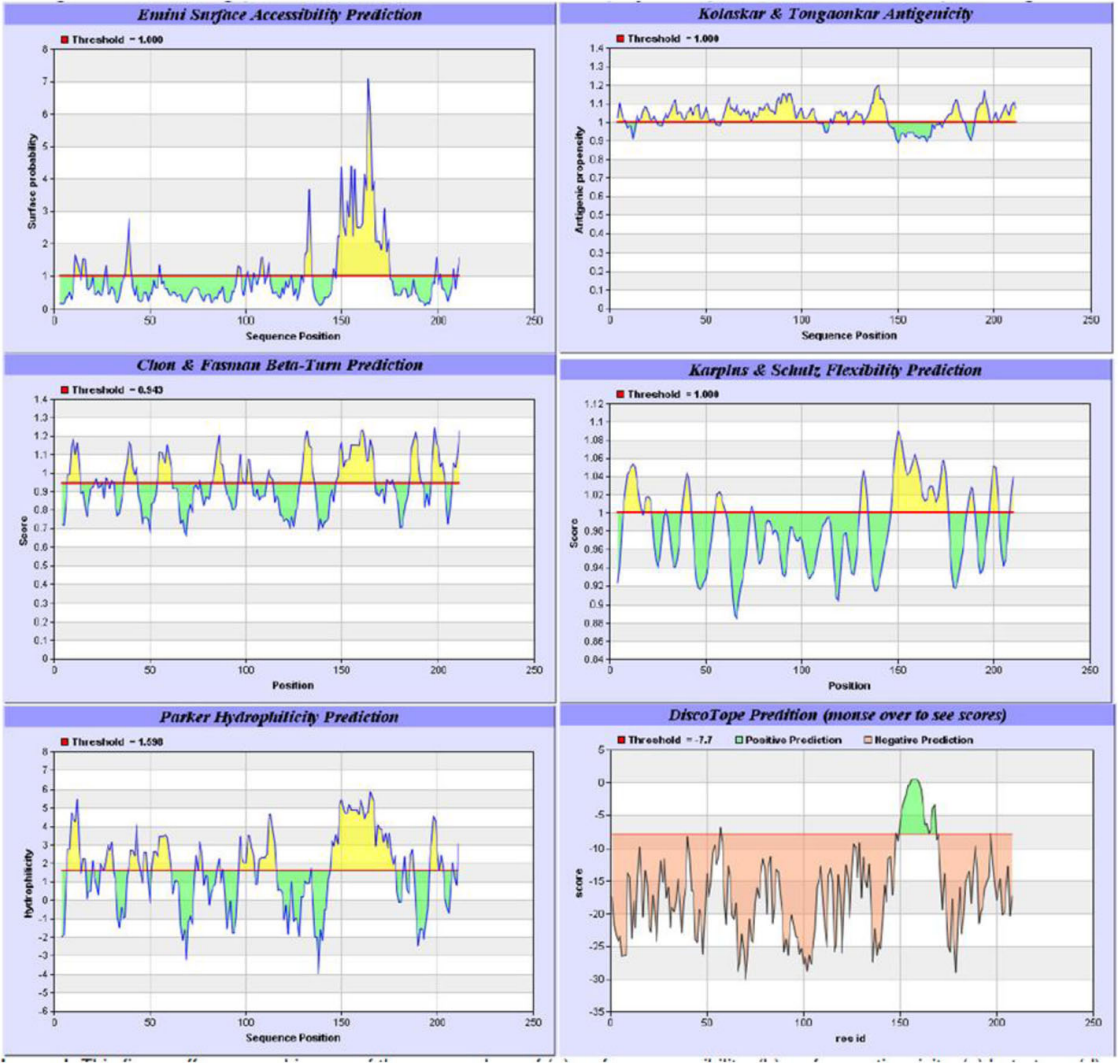
Biophysical Profiles of the TMKmt B cell Epitopes in the Immune Epitope Database Analysis Resource and DiscoTope. This figure shows the 5 biophysical profiles of the linear B cell epitope (UG-Peptide 1) within the IEDB-AR (Plates 1 to 5: surface accessibility, antigenicity, beta-turn, flexibility and hydrophilicity) alongside the DiscoTope profile of the non-linear B cell epitope (UG-Peptide 1)

**Fig. 2:**
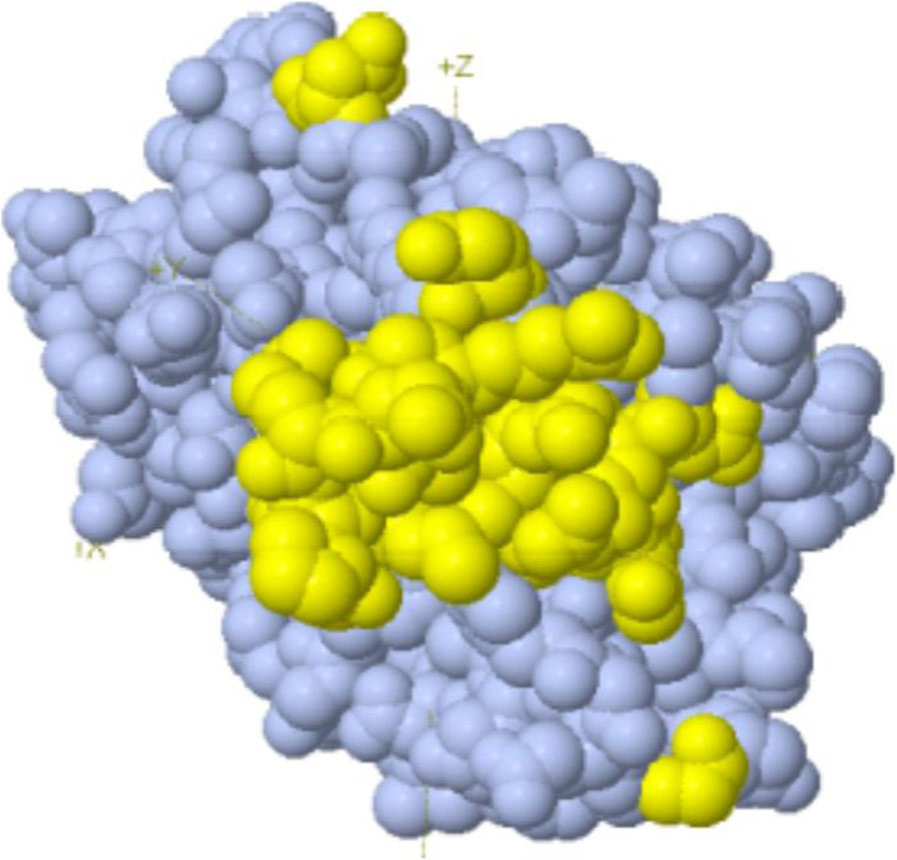
Loci of the non-linear B cell epitope on the 3-D Crystal structure of TMKmt. This figure shows the loci of the 22 amino acids ( A:G57, A:E148, A:S150, A:R151, A:G152, A:R153, A:A154, A:Q155, A:R156, A:D157, A:P158, A:G159, A:A160, A:A161, A:R162, A:A163, A:N164, A:E166, A:R167, A:D168, A:A169, A:T179) on the 3-D crystal structure of TMKmt (PdB entry: “1g3u”).

The identity of the two TMKmt epitopes used to derive polyclonal antibodies (PAb-0655 and PAb-0656) used for TMKmt antigen (Ag) detection Enzyme immuno-assays (EIAs) in this study, was derived  as follows: *First,* (a) using the entire 214 amino acids sequences of TMKmt (SP “|O05891|” ) and five best performing biophysical profiles (accessibility, antigenicity, beta-turn, flexibility, and hydophilicity) in the immune epitope database analysis resource (IEDB-AR), the 27 AA long 148_ERSRGRAQRDPGRARDNYERDAELQQR peptide was predicted to be the best continuous linear B cell epitope by all profiles (4/5, 80%) except antigenicity (see Fig. 1). *Second,* (b) using the crystal structure of TMKmt (PdB entry: “1g3u”) and discontinuous B cell epitope software DiscoTope, we derived 22 amino acids ( A:G57, A:E148, A:S150, A:R151, A:G152, A:R153, A:A154, A:Q155, A:R156, A:D157, A:P158, A:G159, A:A160, A:A161, A:R162, A:A163, A:N164, A:E166, A:R167, A:D168, A:A169, A:T179) as the best discontinuous or non-linear epitope (see Fig. 2). These two epitopes are designated UG-Peptide 1 and 2, respectively. The referenced two synthetic TMKmt-peptide-epitopes and their derivative polyclonal antibodies (PAbs, GeneCUST, Luxemburg) were used to detected TMKmt host specific serum antibody (Ab, IgG) and TMKmt Antigen (Ag) levels by direct enzyme immune-assays (EIA) as previously described [33].

### Methods

#### Design

Cross-Sectional Laboratory Study

#### Site

Immunology laboratory, Dept of Immunology and Molecular Biology, School of Biomedical Sciences, College of Health Sciences, Makerere University Kampala, Uganda.

#### Samples and participants

40 TB exposed house-hold contacts (pre-qualified by QunatiFERON-TB GOLD® as 22 LTBI vs. 18 no TB (NTB); 82 LTBI (46 HIV+ve vs 36 HIV-ve) and 9 NTB.

#### Materials and reagents

TMKmt peptide epitopes 1 and 2 (denoted UG-peptide 1 and 2 respectively, GeneCUST, Luxemburg), New Zealand Rabbit derived anti-UG-peptide 1 polyclonal antibody (Pab-0655); plain ELISA plates (flat bottom, Nunc), Bovine Serum Albumin (BSA, In-vitrogen, USA), goat anti human IgM and IgG (HRP labeled, Bio-Rad, France), Phosphate Buffered Saline (PBS), and the enzymatic substrate tetramethylbenzidine (TMB).

Interventions (a) TMKmt host specific IgM or IgG antibodies: For detection of host TMKmt specific IgM and IgG humoral responses in serum of 40 TB exposed households, we (i) dissolved 1ug (conc: 10mg/ml) of individual synthetic peptide by adding 100 μl of freshly prepared phosphate buffered saline (PBS was prepared by dissolving ¼ of a 250mg tablet in 50ml PCR grade water). (ii) 100 μl (0.001ng) of individual synthetic peptide (UG-Peptide-01 & UG-Peptide-02) was then pipetted into each of the wells of a sterile 96-well microtiter plate (Nunc) and the plate incubated overnight. (iii) The plated wells were then blocked once the following day using 5% BSA in PBS and incubated at 37°C for 30 mins, after which excess solution was discarded and plate left to dry. (iv) 100μls of PBS was added to each assigned wells, followed by addition of 10μl (1:100 dilution) of samples into the respective wells; after which the plate was shaken at 15HZ for 16 secs, and incubated for 1 hour at 37°C. Blank wells were also made, by adding only PBS rather than sample. The wells-in-use were then washed with PBS three times using an automated plate-washer. (v) 100μls of either goat anti-human IgM or IgG horse-raddish peroxidate conjugate was added, and the plates incubated at 37°C for another 1 hour. During this incubation, the enzyme substrate was prepared by adding 1 volume of substrate (TMB) to 1 volume of diluent (hydrogen peroxide) in volumes enough for all the wells in use. (vi) 200μl of freshly prepared substrate was added to each well (purple-bluish color developed in all except A-BX1 blank wells). (vii) The reaction was stopped by adding 100μl of dilute (1mol/L) H_2_SO_4_. The intensity of the reaction in each well was hence after determined by reading the plate at an optical density (OD) of 450nm using a single filter of an automated ELISA plate reader (PR 3100, Bio-Rad). *(b) TMKmt Ag EIA*. For detection of TMKmt Ag among serum of the 82 LTBI categories (i) dissolved 1uL of serum was dissolved in 1000 μl or 1 ml of freshly prepared phosphate buffered saline. (ii) 100 μl of resultant serum-diluent was then pipetted into each of the wells of a sterile 96-well microtiter plate (Nunc) and the plate incubated overnight. (iii) The plated wells were then blocked once the following day using 5% BSA in PBS and incubated at 37°C for 30 mins, after which excess solution was discarded and plate left to dry. Blank wells were also made, by adding only PBS rather than sample. The wells-in-use were then washed with PBS three times using an automated plate-washer. (iv) antipeptide-1 rabbit derived polyclonal antibody (Pab-0655, IgG dominant) was added and plates incubated at 37°C for 30 mins, after which excess solution was discarded and plate left to dry. The wells-in-use were then washed with PBS three times using an automated plate-washer. (v) 100μls of goat anti-rabbit IgG horse-raddish peroxidate conjugate was added, and the plates incubated at 37°C for another 1 hour. During this incubation, the enzyme substrate was prepared by adding 1 volume of substrate (TMB) to 1 volume of diluent (hydrogen peroxide) in volumes enough for all the wells in use. (vi) 200μl of freshly prepared substrate was added to each well (purple-bluish color developed in all except A-BX1 blank wells). (vii) The reaction was stopped by adding 100μl of dilute (1mol/L) H2SO4. The intensity of the reaction in each well was hence after determined by reading the plate at an optical density (OD) of 450nm using a single filter of an automated ELISA plate reader (PR 3100, Bio-Rad).

#### Measured variables

Levels of host specific IgM and IgG antibodies and TMKmt Ag levels in study serum or blanks was qualitatively detected as a function of the OD of each well.

#### Treatment of results

Raw data was cleaned by subtracting ODs of blanks from those of test wells. The issuing adjusted ODs were either run as duplicates in GraphPad® (IgM and IgG) or averaged across the duplicate runs for each test (Ag). Resultant average adjusted ODs were analyzed by both PRISM® software, and Excel® . Graphs were also drawn by GraphPad®. For each OD read (essentially done in duplicate), a 95% Confidence interval (CI) read was computed, alongside the slopes and P-values. Excel sheets were used for correction of average sample OD readings by subtracting OD reading of the blank wells. Only CIs were considered as this exploratory study aimed to demonstrate the accuracy and reliability (replicability) of these assays. Reference to sensitivity, specificity, positive and negative predictive values (PPV and NP as well as receiver operator characteristics (ROCs) curves is made from previous data. To strengthen replicability, testing for Ag capture was done in separate categories of LTBI and results analyzed and presented separate clusters of the same group.

## Results

### IgM and IgG levels among NTB and TB exposed house-hold contacts

#### IgM levels among NTB and TB exposed house-hold contacts

**Fig. 3:**
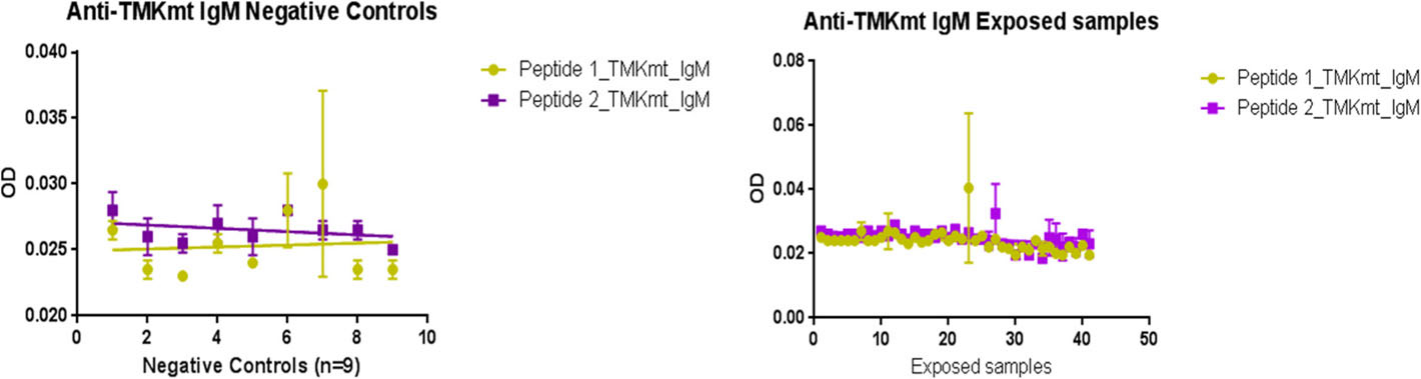
TMK host specific IgM levels among the NTB controls and TB exposed house-hold contacts. This figure shows TMKmt host specific IgM levels among the 9 NTB controls (American donors) and 40 TB exposed house-hold contacts. IgM levels were found to be within the same range across both categories. This is consistent with TB epidemiology and immune-pathogenesis wherein exposure occurs early and is followed by M.*tb* host-specific IgM responses that last only about 1 to 3 months; being replaced by IgG antibodies

TMKmt host specific IgM levels captured by UG-Peptide 1 and UG-Peptide 2 among NTB were 0.02490+0.001599 (95% CI: 0.02151 to 0.02829) and 0.02713+ 0.0006373 (95% CI: 0.02577 to 0.02848) respectively (see Table 1). This when compared to IgM levels captured by the same respective peptide epitopes among TB exposed house-hold contacts of 0.02623+0.0009368 (95% CI: 0.02436 to 0.02809) and 0.02704+0.0006148 (95% CI: 0.02581 to 0.02826) (see Table 2). Overall, these data show that TMKmt host specific IgM levels are similarly low (<0.03) among both NTB and TB exposed household contacts (see Fig. 3). Moreover, there were no observed differences in the capability of either of the two epitopes to capture TMKmt host specific IgM. Therefore, TMKmt host specific IgM levels can not differentiate NTB from LTBI. This is expected, as IgM levels are likely to only be high within 1 to 3 months following the initial exposure to M.*tb* after which they wane and are replaced by IgG levels. It should therefore not be surprising that similarly low IgM levels were found among TB exposed participants. The relevance of TMKmt host specific IgM levels—would thereby be restricted to evaluating risk of exposure to M*.tb* among natives of low TB endemic areas who recently travelled to high TB endemic settings. Indeed, the presence of slightly high levels of IgM among pockets of our purported NTB controls as pre-qualified Quantiferon® Gold assays, might suggest that these persons had inter-currently or recently been exposed to M.*tb*. The latter might actually be a representation of the limitation of QuantiFERON TB Gold ® for differentiating TB exposure from NTB [6–19]. For details, see Additional file 1.

**Table 1:** Showing statistics of TMKmt host specific IgM capture among 9 NTB (duplicates)

	Host Specific IgM	
	UG-Peptide 1	UG-Peptide 2
Best-fit values		
• YIntercept	0.02490	0.02713
• Slope	7.500e-005	-0.000125
Std. Error		
• YIntercept	0.001599	0.0006373
• Slope	0.0002841	0.0001132
95% Confidence Intervals		
• YIntercept	0.02151 to 0.02829	0.02577 to 0.02848
• Slope	-0.0005272 to 0.0006772	-0.0003651 to 0.0001151
Goodness of Fit		
• Degrees of Freedom	16	16
• R square	0.004338	0.07075
• Absolute Sum of Squares	0.0001549	2.463e-005
• Sy.x	0.003112	0.001241
Number of points		
• Analyzed	18	18

**Table 2:** Showing statistics of TMKmt host specific IgM capture among 40 exposed TB house-hold contacts (duplicates)

	Host Specific IgM	
	UG-Peptide 1	UG-Peptide 2
Best-fit values		
• YIntercept	0.02623	0.02704
• Slope	-0.0001115	-0.0001045
Std. Error		
• YIntercept	0.0009368	0.0006148
• Slope	3.905e-005	2.551e-005
95% Confidence Intervals		
• YIntercept	0.02436 to 0.02809	0.02581 to 0.02826
• Slope	-0.0001892 to -3.375e-005	-0.0001553 to -5.377e-005
Goodness of Fit		
• Degrees of Freedom	79	80
• R square	0.09352	0.1735
• Absolute Sum of Squares	0.001368	0.0005975
• Sy.x	0.004161	0.002733
Number of points		
• Analyzed	80	80

#### IgG levels among NTB and TB exposed house-hold contacts

**Fig. 4:**
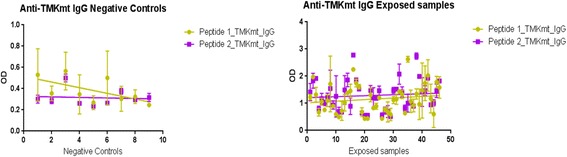
TMKmt host specific IgG levels among the NTB controls and TB exposed house-hold contacts**.** This figure shows TMKmt host specific IgG levels among the 9 NTB controls (American donors) and 40 TB exposed house-hold contacts. Higher IgG levels were observed among the TB exposed house-hold contacts relative to the NTB controls

**Fig. 5:**
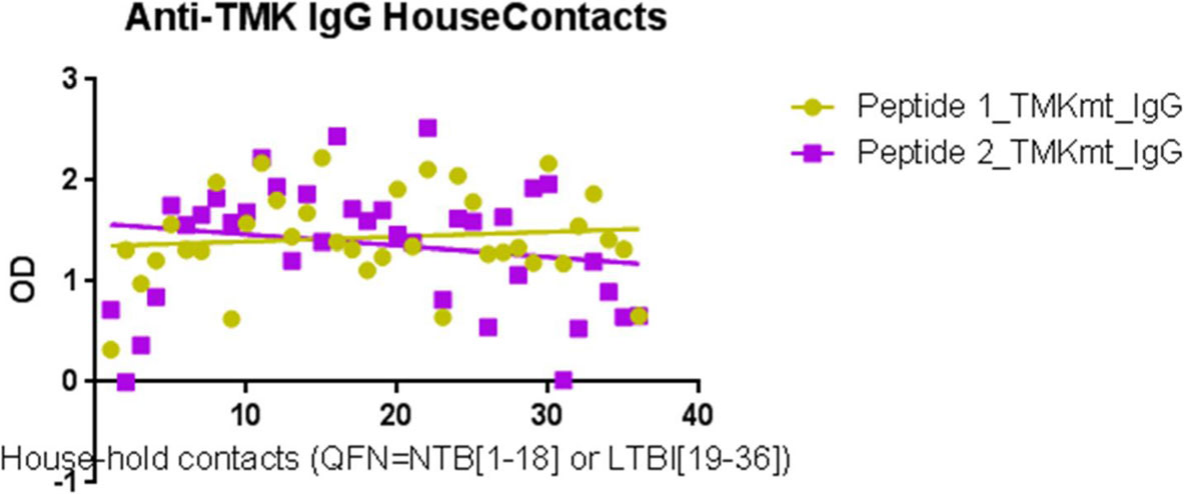
Discordance of distribution of TMKmt host specific IgG levels among the TB house-hold contacts. This figure shows the discordance observed in the distribution of TMKmt host specific IgG levels among the TB house-hold contacts after categorization by QuantiFERON TB Gold® Assay as 18 NTB and 22 LTBI. Considering TMKmt host specific IgG levels, some of the NTB are actually LTBI and vice versa

Unlike the case above, detected IgG levels among NTB controls were in general significantly below those seen among TB exposed house-hold contacts. Specifically, IgG levels captured by UG-peptide 1 and 2 were 0.5155+0.07675 (95% CI: 0.3528 to 0.6782) and 0.3277+ 0.04226 (95% CI: 0.2381 to 0.4173) respectively among NTB controls (see Table 3) compared to 1.020+0.1183 (95% CI: 0.7854 to 1.256) and 1.209 +0.1209 (95% CI: 0.9689 to 1.449) among TB household contacts with LTBI (see Table 4). These trends imply that TMKmt host specific IgG levels are a good potential biomarker for differentiating NTB from LTBI. This data is consistent with the immune-pathogenesis of TB, wherein production of host specific IgG antibody responses to M*.tb* following initial exposure is delayed, but persist once established (see Fig. 4). This data also supports the view that although the designated NTB controls used in this study might not be appropriate by virtue of their measured high IgG levels (due to impreciseness of existing methods for differentiating LTBI from NTB; which is similarly inferred by slightly raised IgM levels among some NTB pointing to perhaps a more recent exposure to M.*tb*), they actually at least offer extremities that are consistent with TB biology. As we shall discuss further, some of the host specific IgG antibody levels seen among the TB exposed house-hold contacts are way above the upper designated limit for LTBI (QuantiFERON TB-Gold®) and qualify to be designated ATB [ATB=OD>1.00; 95% CI1.170 to 1.528 ] as per our prior work [33]. Overall, our prior work supports the view that levels of IgG among true NTB must be lower [=OD<0.088] compared to LTBI [=0.255>OD<1.00]. Indeed, a subsequent attempt to categorize these 40 TB household contacts as NTB and LTBI by the QuantiFERON TB Gold® assay and TMKmt-IgG assay exposes similarly high TMKmt host specific IgG levels among both clusters (see Fig. 5). For details, see Additional file 1.

**Table 3:** Showing Statistics of TMKmt host specific IgG Capture among 9 NTB (duplicates)

	Host Specific IgG	
	UG-Peptide 1	UG-Peptide 2
Best-fit values		
• YIntercept	0.5155	0.3277
• Slope	-0.02662	-0.002550
Std. Error		
• YIntercept	0.07675	0.04226
• Slope	0.01364	0.007510
95% Confidence Intervals		
• YIntercept	0.3528 to 0.6782	0.2381 to 0.4173
• Slope	-0.05553 to 0.002295	-0.01847 to 0.01337
Goodness of Fit		
• Degrees of Freedom	16	16
• R square	0.1923	0.007154
• Absolute Sum of Squares	0.3571	0.1083
• Sy.x	0.1494	0.08227
Number of points		
• Analyzed	18	18

**Table 4:** Showing statistics of TMKmt host specific IgG capture among 40 TB exposed house-hold contacts (duplicates)

	Host Specific IgG	
	UG-Peptide 1	UG-Peptide 2
Best-fit values		
• YIntercept	1.020	1.209
• Slope	0.005842	0.003592
Std. Error		
• YIntercept	0.1183	0.1209
• Slope	0.004384	0.004481
95% Confidence Intervals		
• YIntercept	0.7854 to 1.256	0.9689 to 1.449
• Slope	-0.002867 to 0.01455	-0.005309 to 0.01249
Goodness of Fit		
• Degrees of Freedom	80	80
• R square	0.01935	0.007091
• Absolute Sum of Squares	28.04	29.30
• Sy.x	0.5582	0.5706
Number of points		
• Analyzed	80	80

### TMKmt Ag levels among HIV+ve and HIV-ve LTBI categories *versus* healthy controls

**Fig. 6:**
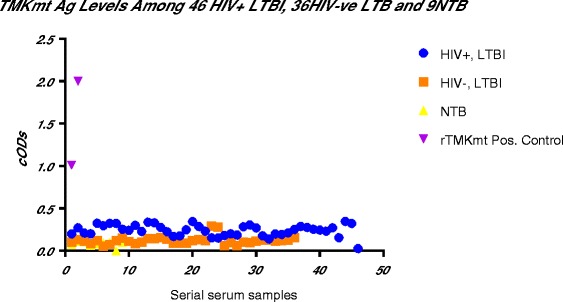
TMKmt Ag levels among HIV+ve LTBI and HIV-ve LTBI relative to NTB controls. This figure shows TMKmt Ag levels among 46 HIV+ve LTBI and 36 HIV-ve LTBI relative to 9 NTB controls. Recombinant TMKmt Ag cloned and expressed in E.*coli* BL21 (DE) was used as a positive control. TMKmt Ag levels among HIV+ve controls where higher than those among HIV-ve LTBI, supporting the high-risk nature of LTBI among PLWHA. Two incidental HIV-ve LTBI had high TMKmt Ag levels, possibly due to false negative HIV-1 test, or another form of immune-deficiency

**Fig. 7:**
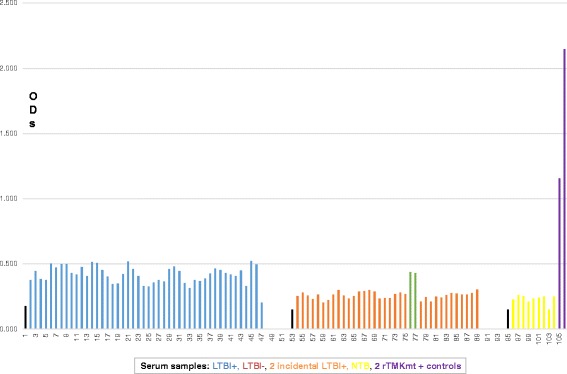
Bar-Graph of TMKmt Ag levels among HIV+ve LTBI and HIV-ve LTBI relative to NTB controls. This figure shows a Bar-Graph of the same TMKmt Ag levels among 46 HIV+ve LTBI and 36 HIV-ve LTBI relative to 9 NTB controls shown in Fig. 6

**Fig. 8:**
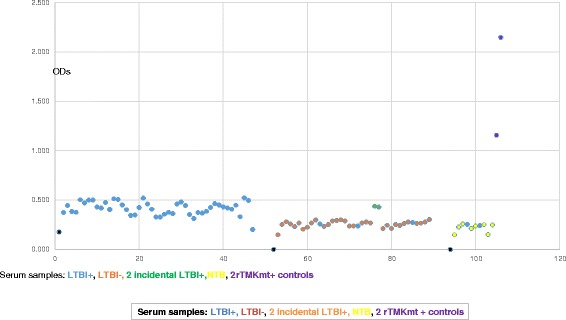
Dot-Graph of TMKmt Ag levels among HIV+ve LTBI and HIV-ve LTBI relative to NTB controls. This figure shows a Dot-Graph of the same TMKmt Ag levels among 46 HIV+ve LTBI and 36 HIV-ve LTBI relative to 9 NTB controls shown in Fig. 6

In order to investigate the above contradictory IgG results in context of the impreciseness of the existing technologies for differentiating between LTBI and NTB, we focused our next evaluations on TMKmt Ag levels. Our earlier work shows that TMKmt Ag levels captured by Pab-655 (derived from UG-peptide 1) can precisely designate ATBI from either LTBI and or NTB [33]. To validate the above findings—we undertook assays of TMKmt Ag levels among an expanded cohort of 82 LTBI (46 HIV+ve vs 36 HIV-ve) and 9 NTB controls. In line with the prevailing knowledge base that LTBI among HIV-1 co-infected is high risk [20–30]; we show that TMKmt Ag levels among HIV+ve LTBI are above those of HIV-ve LTBI. Precisely, it is confirmed that HIV+ve LTBI presents with higher TMKmt Ag levels (>0.14: 0.2676 ± 0.0197 [95% CI: 0.2279 to 0.3073]) relative to HIV-ve LTBI (<0.14: 0.1069 ± 0.01628[95% CI: 0.07385 to 0.14]) (see Table 5). Indeed, as was the case for TMKmt host specific IgG levels, some QuantiFERON-TB Gold® assay pre-qualified NTB had TMKmt Ag levels that lay within ranges of HIV-ve LTBI or epidemiologically low risk LTBI (0.1013 ± 0.02505 [95% CI: 0.0421 to 0.1606]) (see Figs. 6, 7 and 8). Two incidental HIV-ve LTBI had high TMKmt Ag levels, possibly due to false negative HIV-1 test, or another form of immune-deficiency. For details, see Additional file 1.

**Table 5:** TMKmt Ag levels among 82 LTBI categorized as HIV+ve (46), HIV-ve (36) and NTB 9

	HIV+, LTBI	HIV-, LTBI	NTB	rTMKmt Pos. Control
Best-fit values ± SE				Perfect line
• Slope	-0.001101 ± 0.0007299	0.0007494 ± 0.0007674	-0.003667 ± 0.004451	0.991
• Y-intercept	0.2676 ± 0.0197	0.1069 ± 0.01628	0.1013 ± 0.02505	1.5045
• X-intercept	243	-142.7	27.64	-0.01816
• 1/slope	-908	1334	-272.7	1.009
95% Confidence Intervals				
• Slope	-0.002572 to 0.0003697	-0.0008101 to 0.002309	-0.01419 to 0.006859	
• Y-intercept	0.2279 to 0.3073	0.07385 to 0.14	0.0421 to 0.1606	1.009 to 2.000
• X-intercept	117 to +infinity	-infinity to -33.12	10.42 to +infinity	
• Goodness of Fit				
• R square	0.04919	0.02729	0.08837	
• Sy.x	0.06572	0.04783	0.03448	
Is slope significantly non-zero?				
• F	2.276	0.9537	0.6786	
• DFn, DFd	1, 44	1, 34	1, 7	
• P value	0.1385	0.3357	0.4372	
• Deviation from zero?	Not Significant	Not Significant	Not Significant	
Equation	Y = -0.001101∗X + 0.2676	Y = 0.0007494∗X + 0.1069	Y = -0.003667∗X + 0.1013	Y = 0.991∗X + 0.018
Data				
• Number of X values	46	36	9	2
• Maximum number of Y replicates	1	1	1	1
• Total number of values	46	36	9	2
• Number of missing values	0	0	0	0

## Discussion

We present data to support the view that TMKmt Ag level are a potentially more precise and accurate biomarker for incipient LTBI relative to existing assays. Brust B, et al. (2011) recently intimated that those pathogen antigens whose secretory levels depend on the physiology of M.*tb* represent the best candidates for research and development of TB immune-diagnostics [34]. Our group has previously shown that TMKmt Ag levels represent a predictive (foretelling) surrogate biomarker for both *in-vitro* and *in-vivo* growth and proliferation of M.*tb* [33, 35, 36]. Growth and proliferation is a physiologic change that assails M.*tb* exit from dormancy [37–39]. Arguably, there should be more active growth and proliferation of M.*tb* among high-risk LTBI relative to low risk LTBI. Here, using a conceivably novel design of cross-sectional study, we present validation that TMKmt Ag levels are objectively a surrogate biomarker for high-risk LTBI.

*First,* we show similarly low levels of TMKmt host specific IgM (ODs <0.03) captured by EIAs premised on our custom peptide epitopes (see Figs. 1 and 2 respectively) among both NTB and TB exposed household contacts (see Fig. 3 and Tables 1 and 2). For details, see Additional file 1. Exposure to M.*tb* within high TB endemic areas likely occurs immediately following birth [1–5]. As a consequence, IgM levels are bound to only be high within 1 to 3 months following the initial exposure to M.*tb* after which they wane and are replaced by IgG levels. Assays of levels of TMKmt host specific IgM are therefore not usable for differentiating between NTB and LTBI, but might be relevant towards evaluating TB exposure among natives of low TB endemic areas (objectively NTB) who travel to TB high endemic areas and return home within 1-3 months.

*Second,* we show that levels of TMKmt host specific IgG can clearly differentiate NTB from LTBI. Specifically, IgG levels were 0.5155+0.07675 (95% CI: 0.3528 to 0.6782) and 0.3277+ 0.04226 (95% CI: 0.2381 to 0.4173) among NTB controls (see Table 3) compared to 1.020+0.1183 (95% CI: 0.7854 to 1.256) and 1.209 +0.1209 (95% CI: 0.9689 to 1.449) (see Table 4) among LTBI (all captured by the TMKmt epitope peptides UG-peptide 1 and 2 based EIAs, respectively (see Fig. 4). This data is consistent with our prior work that found that levels of IgG among NTB are =OD<0.88 (95% CI: 0.1527 to 0.8751) compared to LTBI=0.255>OD<1.00 (95% CI: 0.2690 to 0.6396) [33]. Indeed, a subsequent attempt to re-categorize the 40 TB exposed household contacts as NTB and LTBI by the QuantiFERON TB Gold® assay alongside the TMKmt-IgG assay revealed high IgG levels among both clusters (see Fig. 5); suggesting that QuantiFERON TB Gold® might be inaccurate towards differentiating LTBI from NTB [3–19]. For details, see Additional file 1. Interestingly, our early work also showed higher TMKmt host specific IgG among HIV+ve persons relative to the HIV-ve persons, a finding we attributed to the myco-septicemia that assails the disseminated nature of TB disease among HIV-1 co-infected persons [33]. These data support the high-risk nature of LTBI among HIV+ve co-infected persons. It is on basis of these and existing epidemiological data that we moved to examine the ability of TMKmt Ag detection as a surrogate for incipient LTBI (assuming that all LTBI among PLWHA is high-risk)[20–30, 33].

In line with the primary hypothesis of this study (that LTBI among HIV+ve persons is high risk)—we show that TMKmt Ag levels among HIV+ve LTBI are above those of HIV-ve LTBI. Specifically, we show that HIV+ve LTBI presents with higher TMKmt Ag levels (>0.14: 0.2676 ± 0.0197 [95% CI: 0.2279 to 0.3073]) relative to HIV-ve LTBI (<0.14: 0.1069 ± 0.01628[95% CI: 0.07385 to 0.14]) (see Table 5). These data support the prevailing WHO & STOP TB Partnership recommendations for isoniazid (INH) prophylaxis among PLWHA within high TB endemic areas [25–30]. As was the case for TMKmt host specific IgG levels, we similarly noted that some QuantiFERON-TB Gold® assay pre-qualified NTB controls had TMKmt Ag levels that lay within ranges of HIV-ve LTBI (0.1013 ± 0.02505 [95% CI: 0.0421 to 0.1606]) (see Figs. 6, 7 and 8). For details, see Additional file 1. Two incidental HIV-ve LTBI had high TMKmt Ag levels, possibly due to false negative HIV-1 test, or another form of physiological or biological immune-deficiency.

In order to offer a hint on the performance of TMKmt Ag and host specific IgG levels for the designation of TB status, we a re-evaluated in-house data from our prior study data for receiver operator characteristics [33]. *On one hand:* (a) for n=128 and prevalence of 80.0 (95% CI: 73.0,86.9), the sensitivity, specificity, PPV and NPV of pre-set TMKmt Ag capture-EIA-OD cut-off for differentiating ATB from NTB alone at 95% CI were respectively**: 99.0** (94.7,100.0), **68.0** (46.5,85.1), **92.7** (86.2,96.8), and **94.4** (72.7,99.9) [yielding a ROC-area of **83.5** (95% CI: 74.1, 92.9)] compared to **96.6** (91.4, 99.1), **21.1** (9.5, 37.3), **78.9** (71.2,85.3) and **66.7** (34.9,90.1) [ROC-area **58.8** (52.0,65.6)] obtained using pre-set TMKmt host-specific IgG-EIA-OD cut-offs on n=154 participants and prevalence of 74.0 (68.0, 81.9). *On the other hand,* (b) for n=220 and prevalence of **63.0** (95% CI: 56.0, 69.1), the sensitivity, specificity, PPV and NPV of pre-set TMKmt Ag capture-EIA-OD cut-off for differentiating ATB from both LTBI & NTB at 95% CI were respectively: **73.9** (65.8,81.0), **90.2** (81.7,95.7), **92.7** (86.2,96.8) and **67.3** (57.7,75.9) [ ROC-area of **82.1** (77.2,87.0)] compared to **92.6** (86.3,96.5), **34.8** (21.4,50.2), **78.9** (71.2,85.3) and **64.0** (42.5,82.0) [ROC-area **63.7** (56.3,71.0)] obtained using pre-set TMKmt host-specific IgG-EIA-OD cut-offs for *n*=167 participants and prevalence of 72.0 (65.0,79.1) (for a summary, see Tables 6, 7 and 8). These sensitivity results are above the documented values for smear microscopy for AFBs (~45%) and close or above those of GenXpert® (72.5 %) [19]. The observed high prevalence rates are as a result of using already pre-qualified specimen rather than undertaking a prospective recruitment and testing.

**Table 6:** Cut-offs values associated with TB status: UG-Peptide 1 based TMKmt host specific Antibody (IgG) capture EIAs (HIV+ve)

TB status	EIA values	95% CI
Active TB (ATB)	ATB=OD>1.00	1.170 to 1.528
Latent M*.tb* Infection (LTBI)	LTBI=0.255>OD<1.00	0.2690 to 0.6396
No TB (NTB)	NTB=OD<0.88	0.1527 to 0.8751

**Table 7:** Cut-offs values for TB status: PAb-655 based TMKmt Antigen capture EIAs (HIV+ve)

Sample TB Status	EIA values	95% CI
Active TB (ATB)	ATB=OD>0.490	0.7446 to 0.8715
Latent M*.tb* Infection (LTBI)	LTBI=0.26>OD<0.490	0.4325 to 0.4829
No TB (NTB)	NTB=OD<0.26	0.1675 to 0.2567

**Table 8:** Receiver operator characterization of TMKmt Ag and host specific IgG EIA-ODs for detecting TB exposure and or disease status among HIV positive individuals

Among HIV positive individuals	Active TB vs OD values classification	
	% (95% CI)	
	ATB vs No TB	ATB vs (LTBI & No TB)
TMKmt Ag Capture		
n	128	220
Prevalence	80.0 (73.0,86.9)	63.0 (56.0,69.1)
Sensitivity	99.0 (94.7,100.0)	73.9 (65.8,81.0)
Specificity	68.0 (46.5,85.1)	90.2 (81.7,95.7)
PPV	92.7 (86.2,96.8)	92.7 (86.2,96.8)
NPV	94.4 (72.7,99.9)	67.3 (57.7,75.9)
ROC area	83.5 (74.1,92.9)	82.1 (77.2,87.0)
TMKmt Ab Capture		
n	154	167
Prevalence	74.0 (68.0, 81.9)	72.0 (65.0,79.1)
Sensitivity	96.6 (91.4, 99.1)	92.6 (86.3,96.5)
Specificity	21.1 (9.5, 37.3)	34.8 (21.4,50.2)
PPV	78.9 (71.2,85.3)	78.9 (71.2,85.3)
NPV	66.7 (34.9,90.1)	64.0 (42.5,82.0)
ROC	58.8 (52.0,65.6)	63.7 (56.3,71.0)

A key limitation of our work—as is the case for all projects that aim to advance novel biomarkers for LTBI, is the absence of a gold standard for precisely designating LTBI and NTB [3–19]. As noted, we maneuvered around this challenge using epidemiological data around LTBI in context of HIV-1 co-infection; and the same design might be relevant to global TB diagnostic biomarker studies [4, 6–14, 40–44, 45–49]. However, the best approach would be a prospective follow-up cohort to recruit high-risk LTBI among HIV+ve persons that eventually develop TB. *Second,* our sample size is small and a larger validation study is needed. Third, much as host immunological and transcriptomic profiles have been studied in an attempt to expose markers of TB disease progression, our group focused on a marker for growth and proliferation of the pathogen (M.*tb*) as a surrogate for disease progression. Although several M.*tb* targets inclusive of lip-arabinomannose (LAM), IP-10, early secretory antigen 6(ESAT-6) and colony filtrate protein 10 (CFP-10) have previously been developed for the purpose of detecting TB, none meets the criteria for designating delineating high-risk LTBI [3–7]. On part of the host, however, recent data report that sequential inflammatory processes and a whole blood RNA signature might possess TB disease progression detection capability that are better than the afore listed M.*tb* targets [50, 51]. It might therefore be worthwhile to adopt assays of TMKmt Ag levels as a new pathogen target for expanded clinical testing towards accurately designating incipient TB. Last but more important to note is that INF-Ƴ responses—which are a correlate of memory, might not present the best biomarker for differentiating between high and low-risk LTBI especially within high TB endemic areas [3–19].

## Conclusion

In conclusion, TMKmt Ag and host specific IgG antibodies offer us novel surrogate biomarkers for LTBI in-context of HIV-1 co-infection. Precisely, Levels of TMKmt Ag represent a novel surrogate biomarker for high-risk LTBI, while host-specific IgG can be used to designate NTB from LTBI. TMKmt host specific IgM levels might be relevant towards evaluating TB exposure among residents of low TB endemic areas (NTB) who recently travelled to a high TB endemic area.

## Additional file

Additional file 1: This file shows processing of the duplicate EIA readings for TMKmt Ag capture among the 46 HIV+ve LTBI and 36 HIV-ve LTBI relative to the 9 NTB controls and 2 rTMKmt positive controls. Overall, readings of blanks were subtracted from the average ODs of the test wells to obtain a corrected average ODs used to draw the graphs. (XLSX 29 kb)

## Abbreviations

Ab: Antibody
Ag: Antigen
OD: Optical Density
EIA: Enzyme Immuno-Assays
M.*tb*: Mycobacterium *tuberculosis*
TB: Tuberculosis
TMKmt: Mycobacterium *tuberculosis* thymidylate kinase
LTBI: Latent *M.tb* Infections
ATB: Active TB
NTB: No TB
